# Coronary Artery Disease and the Profile of Cardiovascular Risk Factors in South South Nigeria: A Clinical and Autopsy Study

**DOI:** 10.1155/2014/804751

**Published:** 2014-03-10

**Authors:** Okon Ekwere Essien, Joseph Andy, Victor Ansa, Akaninyene Asuquo Otu, Alphonsus Udoh

**Affiliations:** ^1^Department of Internal Medicine, University of Calabar Teaching Hospital, PMB 1278, Calabar, Cross River State, Nigeria; ^2^Department of Internal Medicine, University of Uyo Teaching Hospital, PMB 1136, Uyo, Akwa Ibom State, Nigeria; ^3^Department of Chemical Pathology, University of Calabar Teaching Hospital, PMB 1278, Calabar, Cross River State, Nigeria

## Abstract

*Introduction*. Death from coronary artery disease (CAD) has been until recently considered rare in Nigeria. We present a report of a study of CAD with its predisposing cardiovascular (CVD) risk factors in South South Nigeria. *Methods*. We examined the autopsy reports of 747 coroner cases and 41 consecutive clinically diagnosed cases of ischemic heart disease seen in South South Nigeria. *Results*. CAD was diagnosed in 13 (1.6%) of 747 autopsies. They were predominantly males, urban residents, and of high social class with combination of CVD risk factors of hypertension, alcohol use, diabetes mellitus, cigarette smoking, poor physical activities, and obesity. The mean serum cholesterol of the clinical subjects was 4.7 ± 1.57 mmol/L and 5.07 ± 1.94 mmol/L for angina and myocardial infarction, respectively, which was higher than the mean total cholesterol for locality of 3.1 mmol/L. *Conclusion*. CAD and its risk factors are contributing to mortality and morbidity in South South Nigeria. These risk factors include hypertension, alcohol use, diabetes mellitus, cigarette smoking, poor physical activity, and obesity. Nigerians in this locality with CAD have raised serum lipids.

## 1. Introduction

Death from coronary artery disease (CAD) and myocardial infarction (MI) and stroke due to artherosclerosis has attained epidemic proportions in the middle aged and elderly persons in the majority of industrialized countries, accounting for about 50% of all deaths [1, 2]. It has also become the leading cause of death in many developing countries of Asia, the Middle East, and Africa, with increasing incidence [3].

Ischaemic heart disease (IHD) from CAD and its complication, MI, had been shown, by earlier studies done between 1940 and 1970, to be extremely uncommon in Sub-Saharan Africa [4–7]. However, more recent reports indicate that CAD/MI are now more frequently recognized in Nigeria, both clinically and at autopsy, although the incidence is still low compared to that reported in developed countries [8–14].

The established major risk factors that predispose to CAD/MI include hyperlipidemia/dyslipidaemia, systemic hypertension, cigarette smoking, diabetes mellitus, sedentary lifestyle, and obesity [15–17]. Only five of these risk factors have been shown to account for about 89.9% of first MI in Africans. These are current/former tobacco smoking, self-reported systemic hypertension, diabetes mellitus, abdominal obesity measured as waist hip ratio (WHR), and dyslipidaemia [18]. Data from a study shows that Africans who acquire these risk factors either singly or in multiple combinations are at risk of developing CAD and its complications as other people across the globe [19]. The lifestyles of residents of Sub-Saharan Africa, including Nigeria, have significantly been affected by modernization and cultural interaction with Europe, throughout much of the 20th century. The incidence of most of the major CVD risk factors is increasing in most groups of Sub-Saharan Africa, including Nigeria, due to sociocultural and socioeconomic changes resulting in the epidemiological transition the region is passing through [20, 21]. The slow emergence of CAD/MI is probably due to the long incubation period of the disease and may be following on the heels of the progressively changing pattern of CVD risk factors.

In this study, we communicate our observations on the incidence of CAD/MI, using clinical and autopsy cases from South South Nigeria, a region where information on this has been scanty. We also highlight the plausible effects of the low rates of some of the CVD risk factors in the general population that could explain this low incidence thus saving the country the attendant high cost of treatment as observed in the high income countries [22].

## 2. Materials and Methods

This was both a clinical and autopsy study. Subjects were recruited from two Nigerian teaching hospitals, namely, the University of Calabar Teaching Hospital (UCTH) over a fifteen year-period from January 1992 to December 2007 and the University of Uyo Teaching Hospital (UUTH) over a seven-year period from January 2001 to December 2007. Both states are located in the extreme South South zone of Nigeria.

Acute myocardial infarction was diagnosed when typical ischemic chest pain lasted longer than 30 minutes in association with typical ST-T changes on electrocardiography (ECG) and typical cardiac enzymes changes [23]. Established myocardial infarction was diagnosed when a patient with recurrent angina pectoris had a pathological Q-wave on 12 lead ECG and or elevated cardiac enzymes [23].

The participants with angina pectoris were subjected to maximal (symptom limiting) bicycle ergometer stress testing, using modified Bruce Protocol. Only patients with (a) exercise induced angina pectoris, with exercise induced S_3_ gallop, (b) exercise induced ST segment depression ≥ 1.00 mm lasting 0.08 secs and occurring during or immediately after exercise were included in this study [23]. Information on age, sex, occupation, and history of cigarette smoking was obtained. The height (in meters) and the weight (in kilograms) were measured. The body mass index (BMI) was calculated using the formula weight (kg)/height (m^2^). BMI ≥ 30 kg/m^2^ defined obesity. Blood pressure was measured with a standard mercury Accoson sphygmomanometer, with Korotkoff phase 1 determining the systolic blood pressure and Korotkoff phase V determining diastolic BP ≥ 140/90 mmHg defined hypertension. Fasting venous plasma glucose was assayed using glucose oxidase method [24]. The fasting serum cholesterol levels were assayed using enzymatic methods as previously described [25].

The autopsy cases were only recruited in Calabar and involved all coroner cases of sudden death and death from road traffic accidents over an 8-year period. Full autopsies were performed and the hearts examined. History was taken from close relatives and all available medical records were reviewed.

## 3. Results

### 3.1. Autopsy Cases

During the 8-year period (January 1992–December 1999), 747 coroner's cases were performed and 13 (1.6%) of cases of myocardial infarction (MI) were diagnosed. There were 11 males and 2 females, with an age range of 35–63 years; mean age was 51 ± 8.3 and 53.6 ± 12.8 for males and females, respectively.

The mean BMI was 31.36 kg/m^2^. They all belonged to the middle and upper socioeconomic classes. Hypertension was found in 12 (92.3%); 12 (92.3%) also had significant history of alcohol consumption. Eight (61.5%) had diabetes. Eight (61.5%) were cigarette smokers. Five (38.5%) had a combination of HTN, DM, alcohol consumption, and cigarette smoking. One (7.6%) subject had only hypertension but he was also obese and belonged to the upper social class. The majority (84.6%) had anterior wall infarction and 15.4% had anteroseptal wall infarction (See Tables 1 and 2). There were no records of serum cholesterol for these cases.

### 3.2. Clinical Cases

A total number of 110,000 patients were seen in the two study centers over a fifteen-year period. During this period, 41 cases of IHD were diagnosed (21 MI and 20 angina pectoris) giving an incidence of 1 : 2500. There were 34 (82.9%) males and 7 (17.0%) females, with an age range of 26–84 years. Mean ages were 56.7 ± 11.8 and 51.4 ± 12.0 years for males and females, respectively. Majority of the participants were 60 years and above and belonged to the upper socioeconomic class (Table 3).

Sixteen (39.0%) and 15 (36.5%) were hypertensive and obese, respectively. Six (14.6%) had significant alcohol history and 6 (4.4%) reported poor physical activity. Four (9.8%) of all the males smoked cigarettes. Only 2 (4.9%) had diabetes mellitus (Table 4).

Thirty (71.4%) of the participants had serum cholesterol estimated (19 MI, 11 angina). The mean total serum cholesterol of participants with MI was 5.07 ± 1.94 mmol/L and that with angina was 4.73 ± 1.57 mmol/L. The mean HDL was low and triglycerides were high. MI participants had higher total cholesterol, LDL, and triglycerides. However, the differences were not statistically significant (*P* > 0.05) (Table 5).

## 4. Discussion

The findings of this study (autopsy and clinical) show that coronary artery disease is contributing to morbidity and mortality in Nigeria. This is in contrast to the earlier pathologic study that suggested it was rare [7].

In this series of 747 consecutive coroner's autopsies over an 8-year period, 13 deaths were attributed to severe coronary artery disease and myocardial infarction. They had all died suddenly. This is in contrast to earlier pathologic studies which did not find any [5–7]. Though this may represent a rise in incidence of CAD/MI, it is small compared to reports from developed countries. Our observation also supports the findings and conclusions of Ogunnowo and colleagues, that though the incidence of CAD/MI in Nigeria is low, it was rising [8, 9].

Our findings also demonstrate that cases of nonfatal ischaemic heart disease are now being recognized and diagnosed. We recognized 41 participants with MI and angina pectoris over the 15-year period in the two study centers. This number, though small, represents an increase in a geographical area where it has not been previously reported. This pattern of occurrence has also been reported from other parts of Nigeria [8–14]. Ischaemic heart disease is now being diagnosed in Nigeria, albeit infrequently.

The reason for the increase may be related to the changing pattern of disease or the adoption of lifestyles that predispose to the development of cardiovascular risk factors such as hypertension, diabetes mellitus, cigarette smoking, sedentary life style, obesity, and hyperlipidemia [15–17]. The Interheart African study showed that only five of these factors were critical for the development of CAD/MI in Sub-Saharan Africa [18]. These diseases or habits fuelled the epidemic of ischaemic heart disease and its complications in the developed parts of the world [19]. It appears that Nigerians have acquired these risks through exposure to western education and modernization and economic growth and development throughout much of the 21st century [20, 21]. However, a national survey had shown that rates of cigarette smoking and serum lipid levels are low in the general population of Nigerians [26].

The majority of our participants (fatal and nonfatal) were males, middle aged urban residents of the middle/upper socioeconomic classes. This probably suggests less dependence on manual labour compared to their poor peasant rural counterparts. Urbanization promotes remarkable societal and environmental changes that drive the development of IHD and its predisposing risk factors [27]. The incidence of hypertension and diabetes is on the rise in the general population and approximate rates in the USA and Europe [28, 29]. In spite of this, CAD/MI rates in Nigeria are not comparable to rates in USA/Europe [30, 31]. Hypertension more readily causes stroke than ischaemic heart diseases in Nigeria [27]. Hypertension and diabetes mellitus may thus be playing permissive roles.

In Nigeria about 15.4% of adult males and 1.7% of adult females are smokers with more smokers found in the urban than rural areas [26]. This is lower than the current rate of 75% among Americans [32]. Cigarette smoke contains many pharmacologically active agents which damage the vascular endothelium directly and indirectly. These increase the oxidation of low density lipoprotein (LDL), and interact synergistically with hyperlipidemia, hypertension, and diabetes mellitus to promote coronary artherosclerosis [33, 34]. The coexistence of these CVD risk factors in various combinations appears to be critical to the development and progression of CAD. It is important to note that 38.5% of the autopsy cases in the present study had a combination of hypertension, diabetes, cigarette smoking, and more than average consumption of alcohol, in addition to obesity. Eight (61.5%) of these cases of sudden death had a combination of more than three of these CVD risk factors. It thus appears that individuals with more than three CVD risk factors are at increased risk of sudden death attributable to cardiovascular diseases.

Alcohol consumption is a risk factor that has not been previously featured strongly as a modifiable CVD risk factor in Nigeria. Epidemiological studies have shown that mild to moderate alcohol consumption is associated with a reduced risk of development of CAD/MI. Heavy alcohol consumption promotes the progression of artherosclerosis in the carotid artery. Also, binge drinking can trigger a stroke and AMI [35], as 92.3% of fatal cases in the study had a history of excessive alcohol use. This may have contributed to the fatality.

The nonfatal cases shared the same CVD risk profile like the fatal cases. Hypertension and obesity were most frequent, followed by significant alcohol intake and sedentary lifestyle (Table 3). This finding is consistent with reports from other parts of Nigeria [8–14] and globally [19]. Hyperlipidemia appears to play a pivotal role in atherogenesis. This is suggested by animal, genetic, and epidemiological studies. In animal experiments, atherogenic diet that leads to hyperlipidemia increases the incidence of atherosclerosis and atherogenic lesions at all stages and this risk regresses with low lipid/low cholesterol diet and with lipid lowering drugs [36].

Human studies show that hypercholesterolemia occurring in infancy (in homozygous familial hypercholesterolemia) causes myocardial infarction or asymptomatic coronary artery diseases before the age of twenty [36]. Moreover, the death rate from MI/CAD in Japan had been estimated to be 24/100,000, as against 915/100,000 in North Karelia (a butter making and high butter consuming district of Finland), compared with the 299/100,000 Finland national average and 216/100,000 in USA [37]. The fatal cases of CAD in this study had no record of serum lipid levels. However, as they were urban residents and belonged to the middle/upper social classes, obese, or overweight, we suspect that serum lipid levels may have been high. Previous studies have shown that lipid levels are high amongst some Nigerian urban dwellers in the middle/upper social classes [38].

The clinical participants with MI had higher mean serum cholesterol levels than those with angina. Serum lipid levels are generally low in Nigeria. It is 3.1 mmol/L in the forest belt [26] where the present study was carried out. The mean total serum cholesterol of the nonfatal cases in this study was 5.04 ± 1.66 mmol/L and 3.97 ± 2.22 mmol/L for males and females, respectively. This means that Nigerians who develop IHD have serum levels higher than the mean of the general population. The higher the serum cholesterol level, the greater the risk of developing and dying of CAD. This relationship has been demonstrated by the Multiple Risk Factor Intervention Trials (MRFIT), which showed a graded positive relationship between total cholesterol level and CAD mortality as follows: 3.16/1000 for serum cholesterol < 168 mg/dL, (4.31 mmol/L) 4.15/1000 for serum cholesterol 182–192 mg/dL, (4.69–4.92 mmol/L) 5.43/1000 for serum cholesterol 203–212 mg/dL, and (5.20–5.44 mmol/L) 7.35/1000 for serum cholesterol ≥ 264 mg/dL (6.76 mmol/L) [37]. The MRFIT study also showed that the lowest quartile in the USA had serum cholesterol of 5.3 mmol/L as against 4.7 mmol/L for Japan and 4.1 mmol/L for China. The mean serum cholesterol level of our participants with MI was 5.07 mmol/L and 4.73 mmol/L for angina. These values approximate those of the developed countries of USA and Asia where CAD/MI mortality is high. Moreover, the lipid fraction of our participants showed a pattern that promotes atherogenesis, low HDL, and high triglyceride (Table 4).

We observed in this study that the sociodemographic features (male gender, middle age, urban residence, middle/upper social classes) and the behavioural and metabolic risk factors that predispose to CAD/MI in the African are common in the general Nigerian population, except high serum cholesterol levels and low rates of cigarette smoking. These may be protective against the mass occurrence of CAD/MI in this region, at this time.

## 5. Conclusion

Nigeria is at the threshold of epidemiological transition. This is as a result of exposure to western culture, technological developments, rapid urbanization, and acquisition of western lifestyles. This has resulted in the increasing incidence of the risk factors that predispose to atherosclerotic heart diseases. We are now seeing the emergence of CAD/MI in South South Nigeria, a condition hitherto considered as uncommon. We also observed that, of the established major CVD risk factors predisposing to CAD/MI, the prevalence of hyperlipidemia and cigarette smoking is low in the general population of Nigeria. This low frequency of hyperlipidemia may account for the low incidence of CAD/MI in this region of Nigeria, at this time.

Our subjects (autopsy and clinical) who had CAD/MI displayed an array of risk factors predisposing to CAD/MI similar to people in USA/Europe who develop this condition. Our observations in this study and other regions, where CAD/MI are currently uncommon, appear to suggest a pivotal role for the atherogenic diet and the induced hyperlipidemia in the causation of CAD/MI. All other predisposing risk factors appear to induce this disease mainly where there is established hyperlipidemia. The low serum lipid levels may be protective. This is likely to change in the future with a shift in the life style driven by improving socioeconomic status, obesity, and urbanization.

## 6. Limitation

This study was not a case-control study so we could not achieve a head to head comparison of the effect of risk factors. However, this study has demonstrated the increasing prevalence of these risk factors which corroborates reports from other studies.

## Figures and Tables

**Table 1:** The physical, social, and clinical characteristics of the autopsied subjects with MI.

S. number	Name	Age	Sex	BMI	Occupation	Social class	HTN	DM	Alcohol	Cigarette
1	CPM 6/97	63	M	30.7	Custom officer	Middle	+	+	+	++
2	CPM 23/97	48	M	30.1	Politician/BM	Upper	+	−	++	+
3	CPM 27/97	60	M	29.1	Lawyer/BM	Upper	+	+	+	+
4	CPM 2/98	39	F	39.7	International/BM	Upper	+	+	+	−
5	CPM 16/98	55	M	31.1	Trader	Middle	−	+	++	−
6	CPM 29/98	43	M	30.7	Pilot/oil company	Upper	+	−	+	++
7	CPM 34/98	57	M	32.7	RTD school principal	Upper	+	−	−	−
8	CPM 1/99	45	M	34.4	Cocoa merchant	Upper	+	+	++	+
9	CPM 9/99	35	M	28.8	BM	Upper	+	+	+++	++
10	CPM 19/99	59	F	40.6	BM	Upper	+	−	++	−
11	CPM 1/00	60	M	31.7	BM	Upper	+	+	+++	++
12	CPM 9/00	57	M	32.5	Administrator	Middle	+	−	−	−
13	CPM 1/01	55	M	28.5	Administrator	Middle	+	+	−	−

RTD: retired; BM: businessman.

**Table 2:** Sociodemographic and autopsy findings of the coroner's cases.

	Males (*n* = 11)	Females (*n* = 2)	Total (*n* = 13)
Age (years)			
35–39	1 (9.1)	1 (50.0)	2 (15.4)
40–44	1 (9.1)	0 (0)	1 (7.7)
45–49	2 (18.2)	0 (0)	2 (15.4)
50–54	0 (0)	0 (0)	0 (0)
55–59	5 (45.4)	1 (50.0)	3 (23.1)
60 and above	2 (18.2)	0 (0)	2 (15.4)

Total	11 (100)	2 (100)	13 (100)
Mean age (years)	51.5 ± 8.3	53.6 ± 12.8	*P* value (ANOVA) 0.73 (ns)

Social class			
Upper	7 (63.6)	2 (100)	9 (69.2)
Middle	4 (36.4)	0 (0)	4 (30.8)
Lower	0 (0)	0 (0)	0 (0)

Total	11 (100)	2 (100)	13 (100)

Risk factors			
Hypertension	10 (90.9)	2 (100)	12 (93.3)
Diabetes mellitus	6 (54.5)	2 (100)	8 (61.5)
Smoking	7 (63.6)	1 (50.0)	8 (61.5)
Significant alcohol intake	10 (90.9)	2 (100)	12 (93.3)
Obesity	8 (72.7)	2 (100)	10 (76.9)
Autopsy findings *n* = 13			
Anterior wall infarct	11 (84.6)	0 (0)	11 (84.6)
Anteroseptal infarct	1 (7.7)	1 (7.7)	2 (15.4)

ns: not significant (*P* > 0.05).

**Table 3:** Sociodemographic characteristics of clinical participants.

	Males (*n* = 34)	Females (*n* = 7)	Total (*n* = 41)
Age (years)			
35–39	1 (2.9)	2 (28.6)	3 (7.3)
40–44	3 (8.8)	0 (0)	3 (7.3)
45–49	7 (20.6)	2 (28.6)	9 (21.9)
50–54	3 (8.8)	0 (0)	3 (7.3)
55–59	4 (11.8)	1 (14.3)	5 (12.3)
60 and above	16 (47.1)	2 (28.6)	18 (43.9)

Total	34 (100)	7 (100)	41 (100)
Mean age (years)	56.7 ± 12	51.4 ± 11.8	*P* value (ANOVA) 0.289 (ns)

Social class			
Upper	14 (41.2)	4 (57.1)	18 (43.9)
Middle	15 (44.1)	2 (28.6)	17 (41.5)
Lower	5 (14.7)	1 (14.3)	6 (14.6)

Total	34 (100)	7 (100)	41 (100)

**Table 4:** Clinical and biochemical characteristics of clinical cases.

Risk factors	Males (*n* = 34)	Females (*n* = 7)	Total (*n* = 41)
Hypertension	17 (35.3)	4 (57.1)	21 (51.2)
Diabetes mellitus	2 (5.9)	0 (0)	2 (4.9)
Smoking	4 (11.8)	0 (0)	4 (9.8)
Significant alcohol intake	4 (11.8)	2 (28.6)	6 (14.6)
Poor physical activity	3 (8.8)	3 (42.9)	6 (14.6)
Obesity	11 (32.3)	4 (57.1)	15 (36.6)

Fasting lipid levels	Mean ± SD	Mean ± SD	*P* value (ANOVA)

Total cholesterol (Tc)	5.08 ± 1.66	3.97 ± 2.22	0.250 (ns)
HDL	0.87 ± 0.54	0.76 ± 0.52	0.689 (ns)
LDL	3.45 ± 1.22	3.87 ± 1.09	0.5259 (ns)
Triglycerides	1.42 ± 0.53	1.80 ± 0.71	0.232 (ns)

HDL: high density lipoprotein; LDL: low density lipoprotein; numbers in bracket are percentages.

**Table 5:** Comparison of fasting lipid levels between participants with angina pectoris and those with myocardial infarction.

Lipid level	Angina patients	Myocardial infarction	*P* value (ANOVA)
Total cholesterol (Tc)	4.73 ± 1.57	5.07 ± (1.94)	0.605 (ns)
HDL	0.80 ± 0.60	0.91 ± 0.40	0.6239 (ns)
LDL	3.35 ± 1.16	3.67 ± 1.24	0.505 (ns)
Triglycerides	1.46 ± 0.60	1.5 ± 0.55	0.839 (ns)
